# Detection of antibacterial activities of *Miswak*, *Kalonji* and *Aloe vera* against oral pathogens & anti-proliferative activity against cancer cell line

**DOI:** 10.1186/s12906-017-1778-0

**Published:** 2017-05-15

**Authors:** Sameen Amjed, Kashaf Junaid, Junaid Jafar, Tuaha Amjad, Waqas Maqsood, Nadia Mukhtar, Kanza Tariq, Musarrat Sharif, Sana Javaid Awan, Farheen Ansari

**Affiliations:** 1 Institute of Molecular Biology and Biotechnology (IMBB), The University of Lahore, Lahore, Pakistan; 2 Department of University Institute of Medical Laboratory Technology, The University of Lahore, 1km Defence Road off Raiwind road Lahore, Lahore, Pakistan; 3 Fatima Memorial College of Medicine and Dentistry Lahore, Lahore, Pakistan

**Keywords:** Herbal medicine, Antimicrobial, Anti cancer, Antioxidant

## Abstract

**Background:**

Emerging drug resistance and hindrance of treatment is provoking scientists to search new, less expensive medicinally active compounds. Dental diseases caused by oral pathogens are very frequent chronic infections around the world. The medical potentials of a lot of Pakistani local herbs and herbal combinations is relatively unknown, hence attempted to explore. A study was designed to investigate potential role of local medicinal herbs for example *Miswak, Kalonji* & *Aloe vera* as antimicrobial, antioxidant and anti-proliferative agents against oral pathogens and cancer cell line.

**Methods:**

Medicinal extracts were prepared in solvents of different polarities. Their antimicrobial activity was determined alone and in combination against oral pathogens. Antioxidant activity was evaluated through Catalase and Superoxide dismutase assay and anti-proliferative activity was evaluated through 3-(4, 5-Dimethylthiazol-2-Yl)-2,5-Diphenyltetrazolium Bromide) assay.

**Results:**

Plant extracts alone and in combinations were found significantly effective as antimicrobial agent against standard ATCC strains of *C. albicans* and *S. aureus (P* ˂0.001*)*. Especially *Miwak* extract was found highly significant against fungus. Extracts of *Kalonji* were found significant in inhibiting growth of *HeLa* cell lines. *Miswak* and *Kalonji* showed significant levels of antioxidant activity.

**Conclusion:**

Medicinal herbs *Miswak* and *Kalonji* have potential to be used for therapeutic purposes. Results suggested that herbal medicinal composition can be prepared using these extracts after applying scientific standardization methods.

## Background

Since thousands of years herbal products have been the basis for medical treatments and are recognized as a form of alternative medicine. Modern medicine also employs use of many herbal products for therapeutic purposes [1]. The emerging trends of various antimicrobial compounds is alarming; and from last few years researchers are in attempt to search new alternative medicines for treatment of different infectious diseases [2]. Herbal medicines are specially recommended for those patients who cannot use chemical products due to their adverse and biochemical contraindications [3, 4].

World Health Organization (WHO) estimates that 80% of the population in developing countries employs the usage of herbal medicine to treat aspects of primary health care. These medicines also serve as precursor for 20 to 25% of modern medicine as these herbal medicines are considered to be economical, safer and less toxic [5, 6].

Three medically acclaimed plants were included in this study: *Miswak* (*Salvadora persica*) a medicinal herb which is commonly available and very cost effective. It is reported to have antibacterial, antifungal, antioxidant and anticancer properties [7, 8]. *Aloe vera* is a medicinal herb whose mucilaginous gel is traditionally used to treat different diseases. It is very popular among scientists for its medicinal properties [9]. Of the overall weight of *Aloe vera* 2% consist of the active compounds which include methylchromones, flavonoids, aloesin, aloe-emodin, sterols, amino acids, aloemannan, aloin, acemannan, aloeride, naftoquinones, saponin and vitamins. It is considered to be antibacterial, anti-inflammatory and antioxidant [10, 11]. Another important medicinal herb *Nigella sativa* (*Kalonji*) used in this study is reported to possess antimicrobial, anti-inflammatory, antiulcer, anticancer, immune stimulating and antioxidant activity [12–14]. Phytochemical analysis of *Nigella* seeds revealed that it contains a variety of volatile oil and fixed oil and other components including Nigellin, Carvone, Melanthin, Carvene, Cymene and Thymoquinone [14]. A lot of medicinal plants elaborate variety of compounds majority of which have extremely important properties particularly antimicrobial activity and some herbs also possess anticancer activity. Many medicinal herbs are reported to cure different acute and chronic infections including dental infections. Dzoyem in 2016 also have reported antimicrobial activities and anti-cancer activities of fourteen herb against common pathogens [15].

Herbs to herb combinations have been traditionally used in different regions around the word form thousands of years, however scientific evidence based data is still lacking. Traditionally co-administration of herb is believed to influence the overall effect of herb, either complementary or antagonistic [16]. Herbs are believed to contain several potential sources of potent biological compounds. Using herb in combination with others herb or along with antibiotics can produce additional benefits and also reduce the toxicity of herb. Generally herbal combinations are used due to number of reasons as it is believed that their use in combination may give rise to synergistic or additive effect which ultimately helps to overcome drug resistance and increase the spectrum of activity. It may reduce the required dose of administration of any drug and reduce the overall cost along with the side effects [17]. A similar concept in modern medicine is the use of “cocktail” in antiretroviral therapy (HAART) [16]. However less data is present to use these herbs in combinations against oral pathogens. So this study was aimed to ascertain following objectives; to determine antimicrobial activity of these herbs (*Kalonji, Miswak and Aloe Vera*) against oral pathogens, to evaluate anti-proliferative activity against *HeLa* cell line and also antioxidative ability.

## Methods

### Preparation of plant extracts

Samples of under study medicinal plants *Miswak* stem, *Kalonji* seed and *Aloe vera* leaves were collected from local market Lahore after inspecting the organoleptic properties of herbs and final identification was done by herbalist [18]. The preserved herb samples have been submitted with voucher number UOL/PDH627A-C in Herbarium Department of Pharmacy, The University of Lahore. Dried plant samples of *Kalonji* and *Miswak* were crushed to fine particle size using motor pestle. For collecting gel from *Aloe Vera* leaves were freshly cut and washed with distilled water, cuticle was carefully removed and jell was collected [10]. Cold maceration method of extraction was used for the preparation of plant extract in which 200 g crushed plant material was placed in a screw cap container with 600 ml of solvent. Solvents of different polarities were used in this study including petroleum ether, water and ethanol. The herbs macerated in solvents petroleum ether and ethanol was kept in shaker set at room temperature for a period of 7 days. The plant materials macerated in water were kept at room temperature at shaker for 24 hours. Due to the climate conditions it was not possible to macerate water part for 7 days. The *Miswak* sample macerated in petroleum ether was macerated for 21 days because after 7 days very negligible amount of extract was obtained. All the preparations were filtered with Whatman’s filter paper no1 [10, 19]. In all nine filtrates of Petroleum ether *Miswak* (PM), Ethanol *Miswak* (EK), Water *Miswak* (WK), Petroleum ether *Kalonji* (PK), Ethanol *Kalonji* (EK), Water *Kalonji* (WK), Petroleum ether *Aloe vera* (PA), Ethanol *Aloe vera* (EA) and Water *Aloe Vera* (WA) were obtained and shade dried at room temperature. Plant extracts were dissolved in DMSO to obtain 500, 1000 and 2000 μg/disk concentration and subjected to vortex to obtain a homogenized suspension.

### Plant extract in combinations

**Fig. 1:**
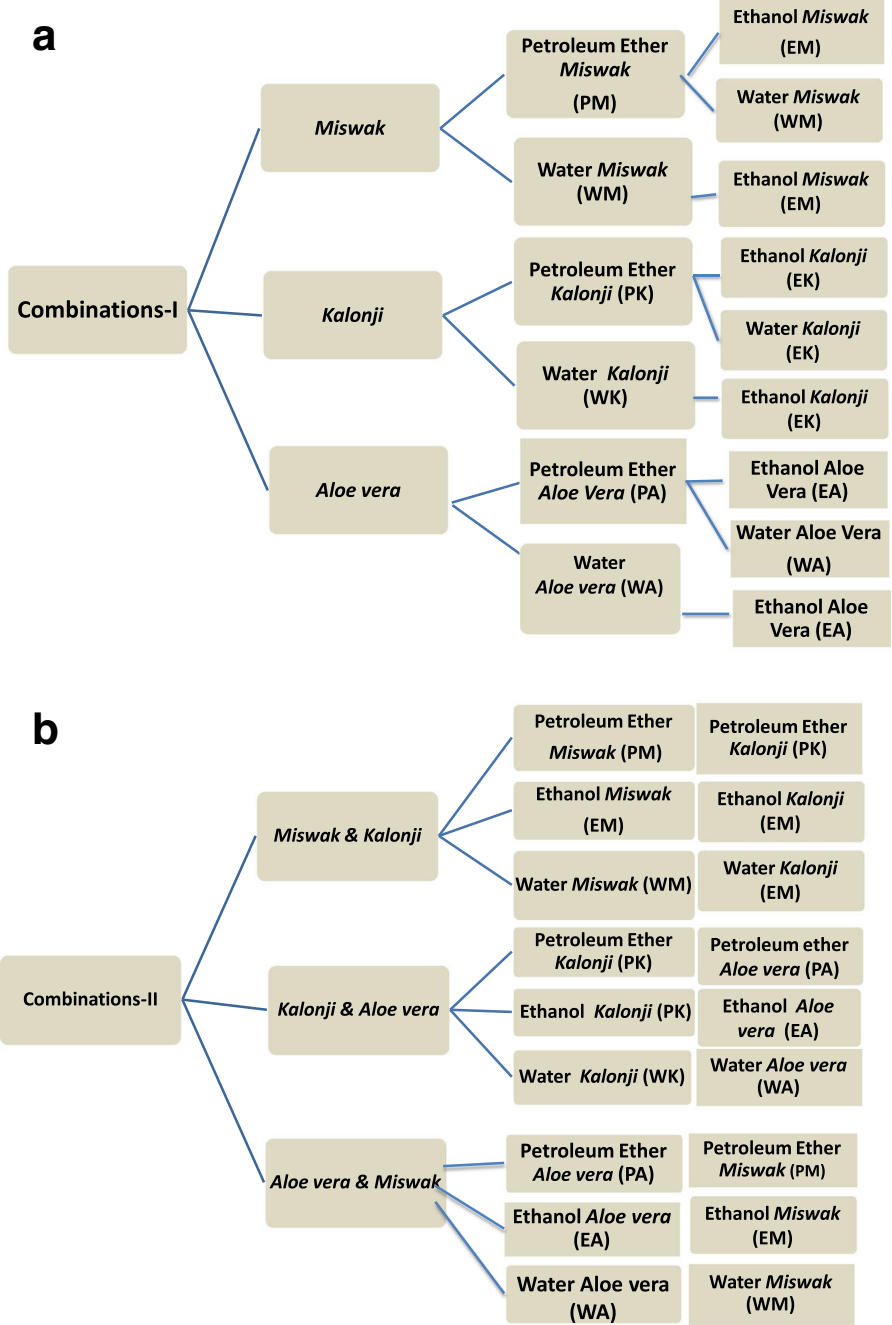
Preparation of herbal extracts in combinations: **a** Combination prepared by mixing extracts of same herb extracted in different solvents (**b**) Combination prepared by mixing different herbal extracts in the same solvent

Eighteen different combinations were prepared (Fig. 1a and b). All combinations were applied at 2000 μg/disc concentration of plant extract. These plant extract in combinations were tested against organisms in triplicate. DMSO was used as negative control, Nystatin 100 IU/disc and Ciprofloxacin 5 μg/disc was used as positive control against *Candida albicans* and *Staphylococcus aureus* respectively.

### Detection of antimicrobial activity

Test organism *Staphylococcus aureus* (ATCC-29213) and *Candida albicans* (ATCC-14053) were further confirmed by their morphological, physiological and biochemical characteristics. The strains were stored at −80 °C in the form of glycerol stocks and were sub cultured at 37 °C for 24 hour; first on nutrient agar and later nutrient broth, before use in susceptibility test. Growth was matched with 0.5 McFarland solutions to prepare the inoculums. Plates of Muller-Hinton agar for bacteria and Sabouraud dextrose agar for fungus were prepared. Bacterial suspension (200 μl volume) was poured and allowed to adsorb over the agar surface. Sterile whatman’s filter paper no1 discs (6 mm in diameter) were freshly impregnated with required concentration of extract (500, 1000 & 2000 μg/disc). However extracts in combination were tested for only one concentration (2000 μg/disc). Ciprofloxacin (5 μg) and Nystatin (100 IU) were used as positive control for *S. aureus* and *C. albicans* respectively while DMSO was used as negative control. These culture plates were incubated at 37 °C for 24 hours. All extracts were run in triplicates and results recorded as zone of inhibition in mm [10]. Minimum inhibitory concentration was determined for those extracts which were producing significant zone of inhibition.

### Treatment of *HeLa* cell line with plant extracts

Stock solution of plant extracts were prepared by dissolving the extracts in DMSO at a concentration of 20 mg/ml. Sterile working solutions of extracts named pre-treatment medium were prepared by the addition of DMEM-HG (Dulbecco’s Modified Eagle’s-High Glucose medium) and the final concentration was1mg/ml [20]. Different group of treatment were named as: a) *HeLa* cells treated with *Miswak* extracts in ethanol (He-T-M-E), water (He-T-M-W) and petroleum ether (He-T-M-PE), b) *HeLa* cells treated with *Kalonji* extracts in ethanol (He-T-K-E), water (He-T-K-W) and petroleum ether (He-T-K-PE), c) *HeLa* cells treated with *Aloe vera* extracts in ethanol (He-T-A-E), water (He-T-A-W) and petroleum ether (He-T-A-PE). The treatment of cell line with plant extracts was continued for 24 hours and after this anti-proliferation and antioxidant activities of medicinal extracts were determined.

### Anti-proliferation activity of plant extracts

To determine anti proliferative activity of treated *HeLa* cells MTT (3-(4, 5-Dimethylthiazol-2-Yl)-2,5-Diphenyltetrazolium Bromide) assay was performed. Treatment medium was removed from micro titer plate and 100 μl of fresh medium was added on the cells. Then 25 μl of MTT solution (5 mg/ml) was added to respective wells and incubated at 37 °C for 2 hours after which purple colored water insoluble formazan crystals appear at the bottom of wells micro titer plate, then 100 μl of 10% Sodium dodecyl sulphate was added to solubilize the formazan crystals for 4 hours. Absorbance (A) was taken at 570 nm. All experiments were performed in triplicate.

### Estimation of antioxidant levels

For the estimation of antioxidant level Superoxide dismutase SOD) and Catalase (CAT) activities were determined in the pretreatment and post treatment medium [21].

### Superoxide dismutase assay (SOD)

Reaction mixture (0.3 ml): 100 mM KH_2_PO_4_ buffer (pH 7.8), 0.1 mM EDTA, 13mMMethionine, 2.25 mM NBT (Nitroblue tetrazolium), 60 μM Riboflavin and plant extracts was prepared. Absorbance was taken at 560 nm after 10 minute of incubation at room temperature.

### Catalase assay (CAT)

To determine Catalase activity reaction mixture (0.3 ml): 1 M KH_2_PO_4,_ 100 mM H_2_O_2_ and plant extract was added in 96 well plate. Absorbance was taken at 240 nm within one min at room temperature.

### Statistical analysis

The results obtained through antimicrobial, anti-proliferative and antioxidant assays were statistically analyzed by SPSS version 20. Descriptive analysis was performed by using Anova test, mean differences of zone of inhibition, anti-proliferative and antioxidant activity verses control was reported with standard error. Figures were drawn by using Graph-pad Prism version 5. Values were considered significant at *P* ≤ 0.05.

## Results

### Percentage yield of medicinal plant extracts

In this study total nine medicinal plant extracts were prepared in solvents of different polarities. The percentage yield of extract obtained from under study crude plant material is given in Table 1.

**Table 1:** Percentage yield of extract

Botanical name	Local name	Plant part used	Solvent	Percentage yield
*Salvadora presica*	*Peelu Miswak*	Stem	Water	2.75%
			Petroleum ether	0.35%
			Ethanol	1.75%
*Nigella sativa*	*Kalonji*	Seeds	Water	0.2%
			Petroleum ether	0.25%
			Ethanol	0.7%
*Aloe vera*	*Kanwar gandal*	Leaves	Water	2.5%
			Petroleum ether	7.5%
			Ethanol	8.75%

### Antimicrobial activity of medicinal plant extracts

**Fig. 2:**
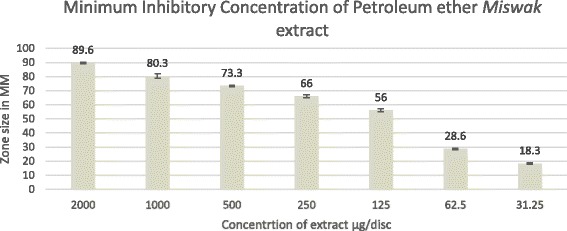
Minimum inhibitory concentration of *Miswak* extracted in petroleum ether against *C. albicans*

The results of this study showed that under studied plant extracts in all solvents were ineffective against *Staphylococcus aureus* at all concentrations used in this study. Similar findings were observed for *Candida albicans* except *Miswak* extract in petroleum ether which showed significant results at all three concentrations (500, 1000 & 2000 μg/disk) and produced zone of inhibition, 73±0.5 mm, 80.5 ± 1.5 mm and 89.6 mm ± 0.5 mm. Minimum inhibitory concentration (MIC) of this extract was 18.3 mm at concentration 31.25 μg/disk (Fig 2).

### Antimicrobial activity of extracts in combinations

When these plants extracts were used in combination they were found very effective as antimicrobial agent against both test organisms and significant inhibitory zones were obtained (*P* <0.001). The minimum and maximum mean zone size produced by extract combinations against *Candida albicans* were 7 mm–66 mm and 7 mm–52 mm against *Staphylococcus aureus*. Of all combinations applied on *Candida albicans* PM in combination with PK gave the maximum zone of inhibition of 66.5± 0.7 mm while EM in addition with WM and PK with PA produced no zone of inhibition. Moreover, three other extract combinations PM+EM, PM+WM and PA+PM produced significant zone of inhibition of 61 ± 0.7 mm, 55 ± 0.7 mm and 65.5 ± 0.7 mm respectively.

**Fig. 3:**
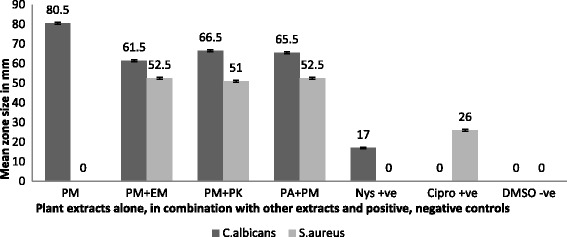
Activity of *Miswak* alone and in combination with *Aloe vera* and *Kalonji* against *S. aureus* and *C. albicans*. Petroleum ether *Miswak* + Ethanol *Miswak* (PM+EM), Petroleum ether *Miswak* + Water *Miswak* (PM+EM), Petroleum ether *Miswak* + Petroleum ether *Kalonji* (PM+PK), Petroleum ether *Aloe vera* + Petroleum ether *Miswak* (PA+PM), Nystatin 100 IU/disc and Ciprofloxacin 5 μg/disc as positive control, and DMSO as negative control

**Fig. 4:**
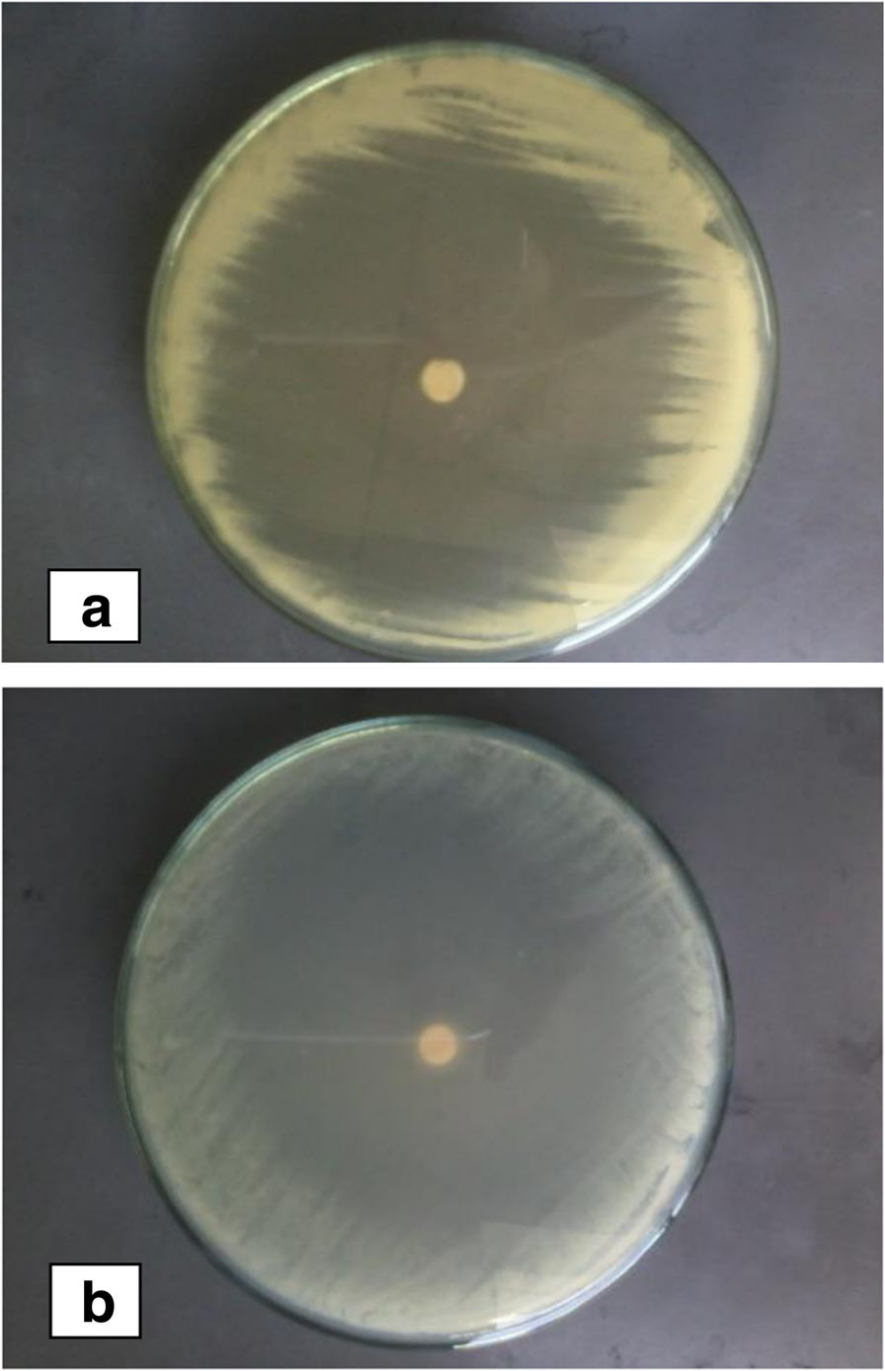
Zone of inhibition produced by *miswak* extract in Petroleum ether alone and in combination with ethanol against both test organisms. **a** PM extract against *c. albicans*, **b** PM+EM extract combination against *S. aureus*

In contrast when extract combinations were tested against *Staphylococcus aureus* PM in addition with EM and PA in addition with PM give the maximum mean zone of 52.5 mm. Likewise extract combination PM with PK and PM with WM also produced significant inhibitory zone of 51 mm and 46 ± 0.00 mm respectively (Fig. 3). Table 2 describes the zone of inhibition produced by all extract combinations against both test organisms. Zone of inhibition produced by *Miswak* extract prepared in petroleum ether (PM) alone and in combination with ethanol (EM) against test organisms is presented in Fig. 4a and b.

**Table 2:** Mean zone of inhibition produced by herbal extract when used in combinations against *S. aureus* and *C. albicans*

Combination	*S. aureus*	*C. albicans*	*P value*
PM+EM	52 mm ± 0.7	61.5 mm ± 0.7	S*
PM+WM	46 mm ± 0	55 mm ± 0.7	S*
EM+WM	0 m	0 mm	NS
PK+EK	9 mm ± 0	8 mm ± 0	NS
WK+PK	8 mm ± 0.7	10 mm ± 0.7	NS
EK+WK	8 mm ± 0	7 mm ± 0	NS
PA+EA	7 mm ± 0	11 mm ± 0.7	NS
WA+PA	0 mm	8 mm ± 0	NS
EA+WA	0 mm	7 mm ± 0	NS
PM+PK	50 mm ± 1.4	66.5 mm ± 0.7	S*
PK+PA	10 mm ± 0.7	0 mm	NS
PA+PM	52 mm ± 0.7	65.5 mm ± 0.7	S*
EM+EK	0 mm	9 mm ± 0	NS
EK+EA	11 mm± 0.7	12 mm ± 0	NS
EA+EM	7 mm ± 0	10 mm ± 0	NS
WM+WK	0 mm	10 mm ± 0.7	NS
WK+WA	0 mm	11.5 mm ± 0.7	NS
WA+WM	0 mm	9.5 mm ± 0.7	NS
DMSO Negative control	0 mm	0 mm	−
Ciprofloxacin Positive control	26 mm ± 0	NA	−
Nystatin Positive Control	NA	17 mm ± 0	−

Petroleum ether *Miswak* + Ethanol *Miswak*(PM+EM), Petroleum ether *Miswak* + Water *Miswak* (PM+EM), Ethanol *Miswak* + Water *Miswak* (EM+WM), Petroleum ether *Kalonji* + Ethanol *Kalonji* (PK+EK), Water *Kalonji*+ Petroleum ether Kalonji (WK+PK), Ethanol Kalonji + Water Kalonji (EK+WK), Petroleum ether Aloe vera + Ethanol *Aloe vera* (PA+EA), Water *Aloe vera* + Petroleum ether *Aloe vera* (WA+PA), Ethanol *Aloe vera* + Water *Aloe vera* (EA+WA), Petroleum ether *Miswak* + Petroleum ether *Kalonji* (PM+PK), Petroleum ether *Kalonji* + Petroleum ether *Aloe vera* (PK+PA), Petroleum ether *Aloe vera* + Petroleum ether *Miswak* (PA+PM), Ethanol *Miswak* + Ethanol *Kalonji* (EM+EK), Ethanol *Kalonji* + Ethanol *Aloe vera* (EK+EA), Ethanol *Aloe vera* + Ethanol *Miswak* (EA+EM), Water *Miswak* + Water *Kalonji* (WM+WK), Water *Kalonji* + Water *Aloe vera* (WK+WA), Water *Aloe vera* + Water *Miswak* (WA+WM). NA: not applicable, S: Significant and NS: Non-significant

S* :Significant

### Anti-proliferative and antioxidant activity of medicinal plant extracts

**Fig. 5:**
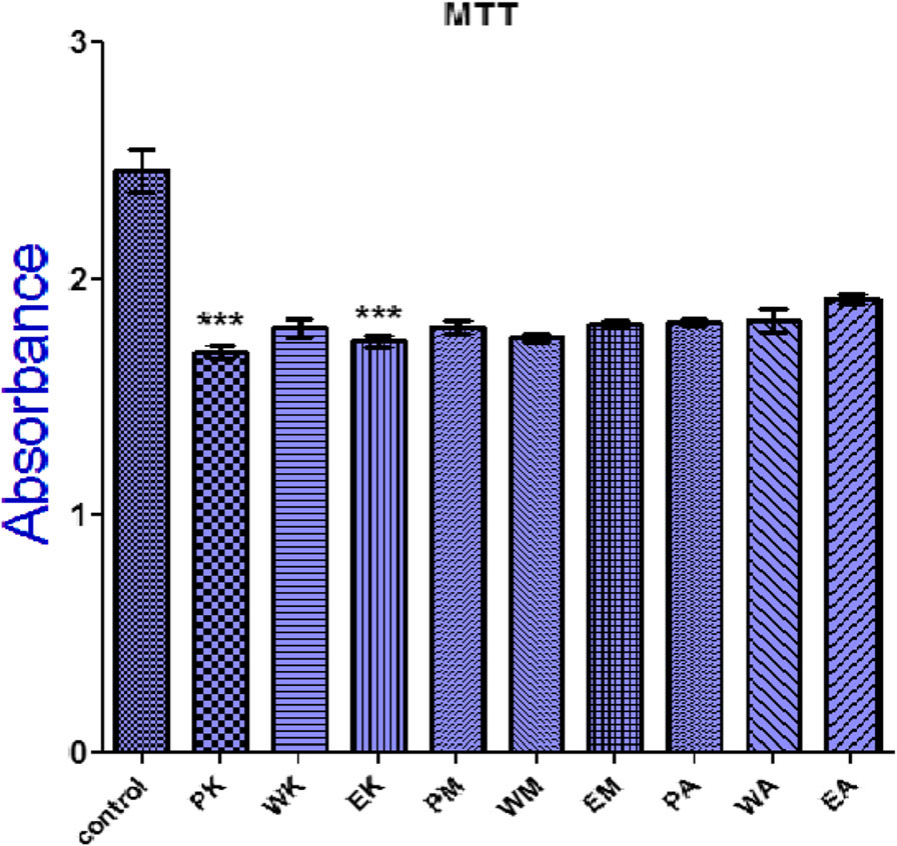
Anti-proliferative activity of plant extract against *HeLa* cell line Note: PM (Petroleum ether *miswak*), EM (Ethanol *Miswak*), WM (Water *Miswak*), PK (Petroleum ether *Kalonji)*, EK (Ethanol *Kalonji*), WK (Water *Kalonji*), PA (Petroleum ether *Aloe vera*), EA (Ethanol *Aloe vera*), WA (Water *Aloe Vera). Absorbance was taken at 570 nm*

The results of MTT assay indicate that significantly low proliferation rate was observed in the presence of two medicinal plant extracts PK (Petroleum ether *Kalonji*) and EK (Ethanol *Kalonji*) (*P* <0.05), which shows the significant difference in the proliferation rate in control cells versus the cells exposed to herbal extracts. The other herbal extracts were not found anti-proliferative against *HeLa* cell line (Fig. 5).

**Fig. 6:**
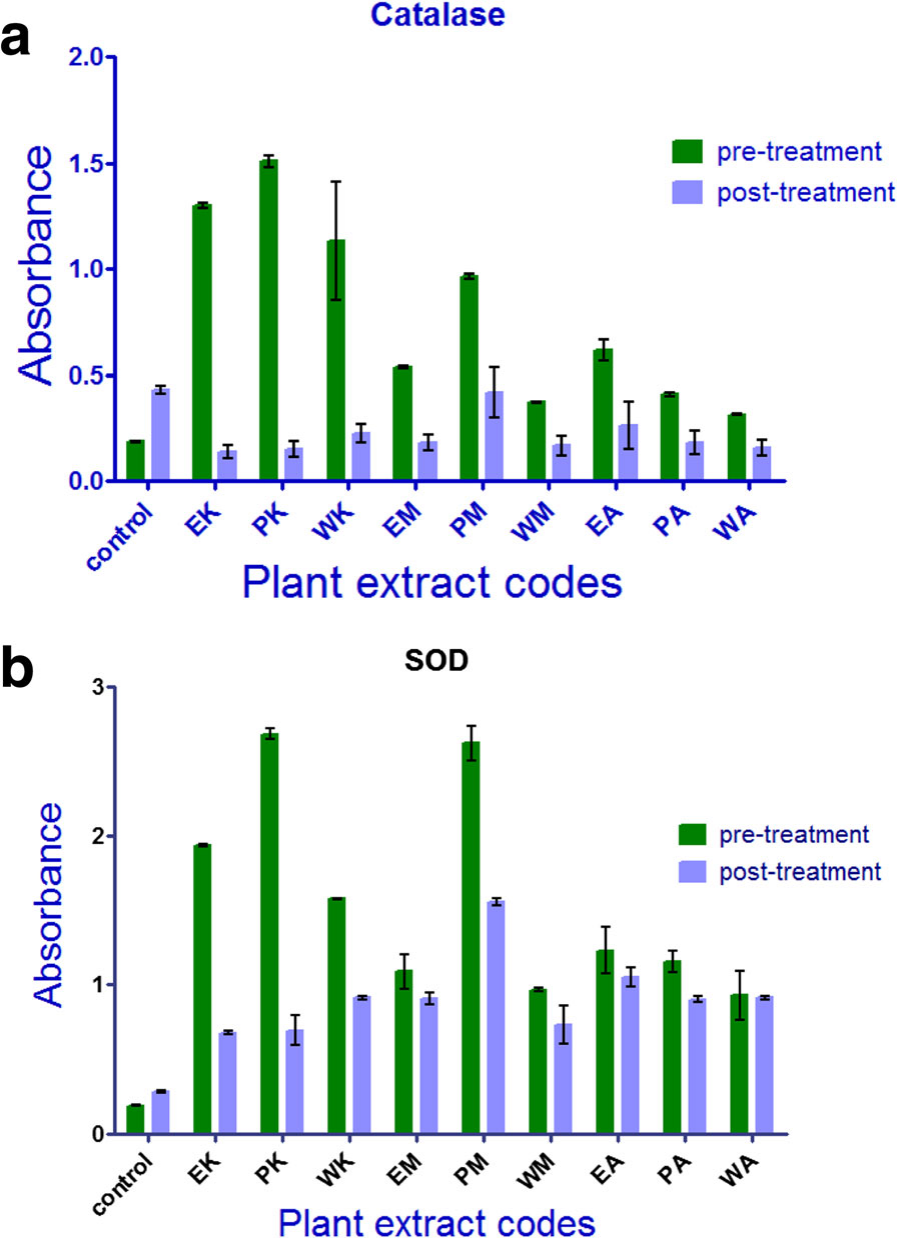
**a** & **b** Catalase and Superoxide dismutase (SOD) activity of herbal extracts Note: PM (Petroleum ether *miswak),* EM (Ethanol *Miswak*), WM (Water *Miswak*), PK (Petroleum ether *Kalonji)*, EK (Ethanol *Kalonji)*, WK (Water *Kalonji*), PA (Petroleum ether *Aloe vera*), EA (Ethanol *Aloe vera*), WA (Water *Aloe Vera*). The figure shows the pretreatment and post treatment levels of Catalase and Superoxide dismutase levels of herbal extract verses controls with standard error. The absorbance was taken at 240 nm and 560 nm respectively

The results of Catalase and Superoxide dismutase antioxidant assays shows that four herbal extracts EK (Ethanol *Kalonji*), PK (Petroleum ether *Kalonji*), WK (Water *Kalonji*) and PM (Petroleum ether *Miswak*) showed significant level antioxidant activity (*P* <0.001) indicates significance of difference between the pre-treatment and post-treatment antioxidant ability of these herbal extracts as presented in Table 3. All other extracts including EM, WM, EA, PA and WA showed no significant difference between the Catalase and SOD levels of pre-treatment and post-treatment samples of herbal extracts (Fig. 6a and b).

**Table 3:** Pre and post-treatment of catalase and superoxide dismutase activity of herbal extracts

Assay	Extract	Pre-treatment	Post-treatment	*P value*
Catalase (CAT)	Control	0.19	0.43	S*
	EK	1.3	0.14	S*
	PK	1.5	0.15	S*
	WK	1.1	0.22	S*
	EM	0.54	0.18	NS
	PM	0.96	0.42	NS
	WM	0.37	0.17	NS
	EA	0.62	0.26	S*
	PA	0.41	0.18	S*
	WA	0.32	0.16	S*
Superoxide dismutase (SOD)	Control	0.19	0.28	S*
	EK	1.9	0.68	NS
	PK	2.7	0.69	NS
	WK	1.6	0.91	NS
	EM	1.1	0.91	S*
	PM	2.6	1.6	NS
	WM	0.96	0.73	S*
	EA	1.2	1.1	S*
	PA	1.2	0.90	S*
	WA	0.93	0.91	S*

Ethanol *Kalonji* (EK), Petroleum ether *Kalonji* (PK), Water *Kalonji* (WK), Ethanol *Miswak* (EM), Petroleum ether *Miswak* (PM), Water *Miswak* (WM), Ethanol *Aloe vera* (EA), Petroleum ether *Aloe vera* (PA) and Water *Aloe vera* (WA). Values represented as absorbance at 240 nm for Catalase assay and at 560 nm for Superoxide dismutase respectively. “*NS*” non-significant and “*S*” significant. Values were considered significant at *P* ≤ 0.05.

S* :Significant

## Discussion

The results of this study indicate that medicinal herbs *Miswak, Kalonji* & *Aloe vera* contain significant level of antimicrobial, antioxidant and anti-proliferative agents in them and hence needed to be further explored. Production of synthetic drugs effectively improves the health care facilities around the world. In developing countries still local herbal products are common to treat a lot of diseased conditions due to their cost effectiveness and less side effects [22]. Fabricant & Farnsworth in 2001 reported active compounds obtained from herbal products have 80% correlation between their traditional use and use in modern therapeutics [16, 23]. Rath S and Padhy RN in 2015 stated that therapeutic plants have immense potential to be used in integrative and synergistic drug development [24].

Test organisms in this study *Staphylococcus aureus* and *Candida albicans* are the most common cause of oral infections across the world [25, 26]. Our findings of herbal extracts affectivity against oral pathogens are in consistence with previous reports [7, 8, 27]. Al-Obaida and coauthors declared *miswak* as an effective oral hygienic tool and found it helpful against different organisms causing oral cavity infections [28, 29]. A study conducted by Heshama A.E 2016 stated that the beneficial effects of *miswak* on oral health are due to presence of different important minerals and compounds. The combination of Benzyllisothiocyanate, salvadorine, fluoride, tannins, vitamin C, Silica, chloride and essential oils contribute to the value of *miswak* [30].

The results of this study are comparable with the previous studies as the prepared (PM) *Miswak* extract produced significant zone of inhibition against *Candida albicans* but contrary to this aqueous and ethanol extracts were found ineffective when applied on both test organisms. Another study showed that aqueous and ethanolic extracts of *Miswak* produced significant zone of inhibition against *Staphylococcus aureus* and *Candida albicans* [27]. Salehi et al. also showed that *Miswak* extracts used in form of mouthwash were effective against oral pathogens [29]. Another study reported reduction w?>in number of organism by the use of *Miswak* extracts as mouth wash [27]. Omer et al. stated that *Miswak* extracts when prepared in solvents chloroform, ethyl acetate and ethanol it produced no zone of inhibition against *Candida albicans* and *Staphylococcus aureus* but its aqueous extract was effective [31]. In contrast to Al-Obaida et al. reported herbal ethanol extracts were effective against *S. aureus and C. albicans*. In 2013 Bafti et al. reported *Miswak* extracts contain good antifungal properties [3]. Finding of our study also suggest *Miswak* as an antioxidant agent it contain antioxidant compounds for example peroxidase, catalase and polyphenol oxidase representing it as a good chewing stick [7].

These inconsistent findings of *miswak* extract are might be due to the difference in the environmental, climate, and harvesting conditions of herbs. The results of our study show some difference as compared to the published data. But there are inconsistent reports regarding herbal activities. These variation in the activity of herb results are comparable with other studies including Teka et al. [32], Darout et al. [22], Bakathir and Abbas [33], Togan et al. [18], Khan et al. [14]. Findings of these authors suggest that variation in results of herbs when applied against organism can come due to many reasons for example geographical location, harvesting conditions, chemical constituents and use of different techniques of extraction. Activity of the herb also varies if the organism is isolated from clinical samples or standard strains [18, 22, 32–34].

*Aloe vera* being a phytotherapic agent also act as inhibitory factor for the growth of wide range of oral microorganisms and is reported to significantly reduce intensity of gingivitis and plaque formation [35, 36]. However, in this study *Aloe vera* extracts did not exhibit antimicrobial activity in consistent with previous studies where it’s aqueous and methanol extracts were found effective against *Candida albicans* but were ineffective against *Staphylococcys aureus* [37].

*Kalonji* also called miracle herb due to its medicinal properties. It is a commonly grown herb all over the world. In herbal medicine its seeds are commonly used to treat many diseases [14, 38]**.** Many studies reported it’s antioxidant, antibacterial, antifungal, anti-plaque and anti-proliferation activities [14]. The results of our study also showed similar findings as extracts prepared from *Kalonji* showed significant antioxidant and anti-proliferative abilities especially when extracted in ethanol and petroleum ether but not antimicrobial activity. A study conducted by Khan et al. 2013 in Pakistan also show comparable result to our finding where *Nigella* extracted in methanol was found ineffective when applied against *S. aureus*. Studies also suggested that biologically active medicinal plants can show inhibitory activity against some organism but can be ineffective against others [34].

Our finding that extracts in combinations produce significant antimicrobial activity, when applied on both test organisms *(P* <0.001*)* is striking. In some extracts when they were tested as alone extracts were not active against *Staphylococcus aureus* while when tested in combinations with other extracts significant zone of inhibitions were produced this may be attributed by the synergistic and antagonistic effect produced by constituents of herbal extract [39, 40].

The study conducted by Bag et al. in 2015 also enforce that when two herbs are given in combination the biological effects of herbal combination is enhanced and show synergistic effect [17]. This synergistic effects are due to the interrelationships of herbal ingredients where synergy can be broadly classified into two main categories based on the mode of actions including pharmacodynamic (when two or more constituents work on same biological targets and result in improved therapeutic outcomes) and pharmacokinetic (two or more constituents interactions between their metabolic processes, absorption, distribution and elimination) [41].

This study was designed with the hypothesis that medical herbs contain potent antitumor and anti cancerous agent. We examined the effect of *Kalonji, Miswak* and *Aloe vera* extracts in petroleum ether, ethanol and water at a concentration of 1 mg/ml. Findings suggest only *Kalonji* extracted in petroleum ether and in ethanol have anti-proliferation activity with a statistical border line significant *p value*. These results are in agreement with previous studies already reported anti proliferative and antioxidant activity of herbs [20, 27, 29].

As the effectiveness of these herbs in combination have not been previously explore that provides this study more strength. However, there are few limitations of this study; due to limited availability of extract it was not possible to test herbal combinations at different concentrations and it was also not possible to make further herbal combinations so only 18 different combinations of extracts were used. This small volume of our extracts also made it impossible to test anti-proliferative activity on other cell lines this leads to uncertainty in these results. However it is recommended to further check the activities of these herbs on other cell lines.

This study suggests medicinal herbs particularly *Miswak* and *Kalonji* have potential to be used for therapeutic purpose. These medicinal herbs should be tested in further combinations to determine their medicinal potentials. *Miswak’s* therapeutic efficacy can be increased in many folds in combination with other medicinal herbs hence it should be further investigated. More research work should be conducted in the field of phytochemistry focusing on both in vitro and in vivo analyses and the isolation of bioactive compounds of interest [42].

## Conclusion

In light of this study it can be concluded that herbs *Kalonji, Miswak* and *Aloe vera* were demonstrated to contain significant level of antimicrobial, anti-proliferative and antioxidant agents in them. It has been proved in this study that when two different herbs are used to evaluate antimicrobial activity the component of one herb dramatically changed the activity of other herb. The results of our study also revealed this intra-component activity of herb to herb combination (*Kalonji* and *Aloe vera*) when used alone against *S. aureus* showed ineffective result but a synergistic effect on antibacterial activity of these herbs has been observed when used in combination with *Miswak* in the same solvent. The optimum activity of herb was achieved when extracted in petroleum ether.

## Abbreviations

EA: Ethanol *Aloe vera*
EK: Ethanol *Kalonji*
EM: Ethanol *Miswak*
PA: Petroleum ether *Aloe vera*
PK: Petroleum ether *Kalonji*
PM: Petroleum ether *miswak*
WA: Water *Aloe Vera*
WK: Water *Kalonji*
WM: Water *Miswak*
